# The impact of health literacy in the care of surgical patients: a qualitative systematic review

**DOI:** 10.1186/s12893-015-0073-6

**Published:** 2015-07-17

**Authors:** Gildasio S. De Oliveira, Robert J. McCarthy, Michael S. Wolf, Jane Holl

**Affiliations:** Department of Anesthesiology, Feinberg School of Medicine, Northwestern University, 241 East Huron St, F5-704 Chicago, IL USA; Department of Medicine, Feinberg School of Medicine, Northwestern University, Chicago, USA; Center for Health Care studies, Northwestern University, Chicago, USA

## Abstract

**Background:**

Inadequate health literacy affects more than 90 million Americans and it has been associated with adverse outcomes in the medicine field including increased hospitalization rates and greater mortality. Since surgical patients are often required to make complex decisions and adhere to complex instructions, health literacy may have a profound impact in the surgical practice. The main objective of the current study was to systematically evaluate the role of health literacy in surgical patients.

**Methods:**

A systematic search was performed to identify studies that evaluated the role of health literacy in the perioperative setting following the PRISMA guidelines. Only studies that examined health literacy using a validated instrument in the perioperative setting were included.

**Results:**

Ten studies including data on 1147 patients were included. The median (IQR) number of patients in the included studies was 101 (30 to 152). The majority of studies used the Short Test of Functional Literacy in adults (STOFHLA) to evaluate patients’ health literacy. Five studies evaluated the patients preoperatively, four studies evaluated patients in the postoperative period and in one study the time of evaluation in relation to the surgical procedure was not defined. The lowest prevalence of inadequate health literacy was detected in kidney transplant patients, 6 out of 124 (5 %), while the highest prevalence of inadequate health literacy was detected in orthopedic patients having total joint replacement, 86 out of 126 (60 %). Inadequate health literacy in the preoperative period was associated with poor medical information comprehension and it may adversely affect adherence to preoperative medications and even modulate surgical disparities. Inadequate health literacy in the postoperative period was associated with poor comprehension of discharge instructions and worse kidney function in transplant recipients.

**Conclusions:**

Health literacy seems to have a very significant impact in the care of surgical patients. More studies to establish the impact of poor health literacy on perioperative outcomes are needed.

## Background

Health literacy is the ability to comprehend and use health information in order to make appropriate health decisions [1]. It has been estimated that over 90 million Americans have inadequate health literacy [2], that makes them unable to understand basic instructions and make appropriate health related decisions [3]. In non-surgical patients, several large studies have demonstrated an association between poor health literacy and negative patient outcomes such as increased hospitalizations and greater mortality [4, 5]. In addition, poor health literacy has been estimated to cause an economic burden to the health care system of approximately $75 billion per year in the US alone [6].

Health literacy is likely to have a very important role in the care of perioperative patients. In addition of an understanding of the health information they are provided, numeracy skills are required to make decisions regarding elective surgical procedures. Frequently, patients are required to follow complex preoperative and postoperative instructions and lack of adherence to those instructions can result in negative outcomes or cancellation of surgical procedures [7, 8]. Despite the established role of health literacy and outcomes in non-surgical patients, the role of health literacy in surgical patients is currently not well defined.

The main objective of the current systematic review was to evaluate health literacy in surgical patients. We also sought to examine the current reported prevalence of patients with inadequate health literacy in the same population.

## Methods

We performed a quantitative systematic review following the guidelines of the Preferred Reporting Items for Systematic Reviews and Meta-Analyses statement (PRISMA) [9]. The current manuscript is a review article and does not require IRB approval.

### Systematic search

Published reports of studies evaluating health literacy in the perioperative setting were searched using the National Library of Medicine’s Pubmed database, the Cochrane Database of Systematic Reviews and Google Scholar inclusive to November 15^th^, 2013. Free text and MeSH terms ‘literacy’, ‘health’, ‘surgery’, ‘perioperative’, ‘preoperative’, ‘postoperative’ and ‘operation’ were used individually and in pairwise combinations. No language restriction was used. An attempt to identify additional studies not found by the primary search methods was made by reviewing the reference lists from identified studies. No search was performed for unpublished studies. This initial search yielded 291 manuscripts.

### Selection of included studies

The study’s inclusion and exclusion criteria were determined before the systematic search. Two authors (GDO and RJM) independently evaluated the abstract and results of the 291 articles obtained by the initial search. Articles that were clearly not relevant based on our inclusion and exclusion criteria were excluded at this phase. Disagreements on inclusion of the articles were resolved by discussion among the evaluators. If an agreement could not be reached, the dispute was resolved with the help of a third investigator. The third investigator was blinded regarding evaluation of the first two authors.

### Inclusion and exclusion criteria

We included published manuscripts that evaluated health literacy in perioperative patients. Included studies had to report on health literacy using a validated instrument. Inclusion of studies was not limited by the timing of healthy literacy evaluation in relation to the surgical procedure (preoperative or postoperative). Studies that examined topics related to health literacy but did not include reports on patients were excluded. Excluded also were studies that performed simple readability tests of patient education materials. No minimum sample size was required for inclusion in the systematic review.

### Validity scoring

The Newcastle-Ottawa scale was used to assess the methodological quality of cohort and case–control studies [10]. The Newcastle-Ottawa scale contains eight items that are divided in three sections: selection (four items), comparability (one item) and exposure (three items). A star is given to each category that presents a high-quality choice of individual study. For randomized trials a modified Jadad five point quality scale was used to assess study quality. The scale evaluates the study for the following: randomization, double blind evaluation, concealment of study group to evaluator, valid randomization method and completeness of data at follow-up [11]. Two authors (GSD and RJM) independently read the included reports and assessed their methodological validity. Discrepancies in rating of the trials were resolved by discussion among the evaluators. If an agreement could not be reached, the dispute was resolved with the help of another investigator. Studies were not excluded or weighted in the analysis based on quality assessment scores.

### Data extraction

Two authors (GDO and RJM) independently evaluated the full manuscripts of all included studies and performed data extraction using a data collection form specifically developed for this review.

Discrepancies were resolved by discussion between the two investigators (GDO and RJM). If an agreement could not be reached between the two investigators, the decision was made by another investigator. Data extracted from studies included the health literacy instrument used, type of surgical specialty, time in relation to surgical procedure (preoperative, intraoperative or postoperative), type of intervention, evaluated outcomes, study design, sample size, number of subjects with inadequate literacy, and follow-up period.

Data were initially extracted from tables or text. For data not available in tables, the data was abstracted from available figures. Dichotomous data on the presence or absence of adverse effects was extracted and converted to incidence, while continuous data was recorded using mean and standard deviation.

### Meta-analyses

Quantitative analysis was not performed due to the large heterogeneity of study designs, interventions and measured outcomes.

## Results

**Fig. 1:**
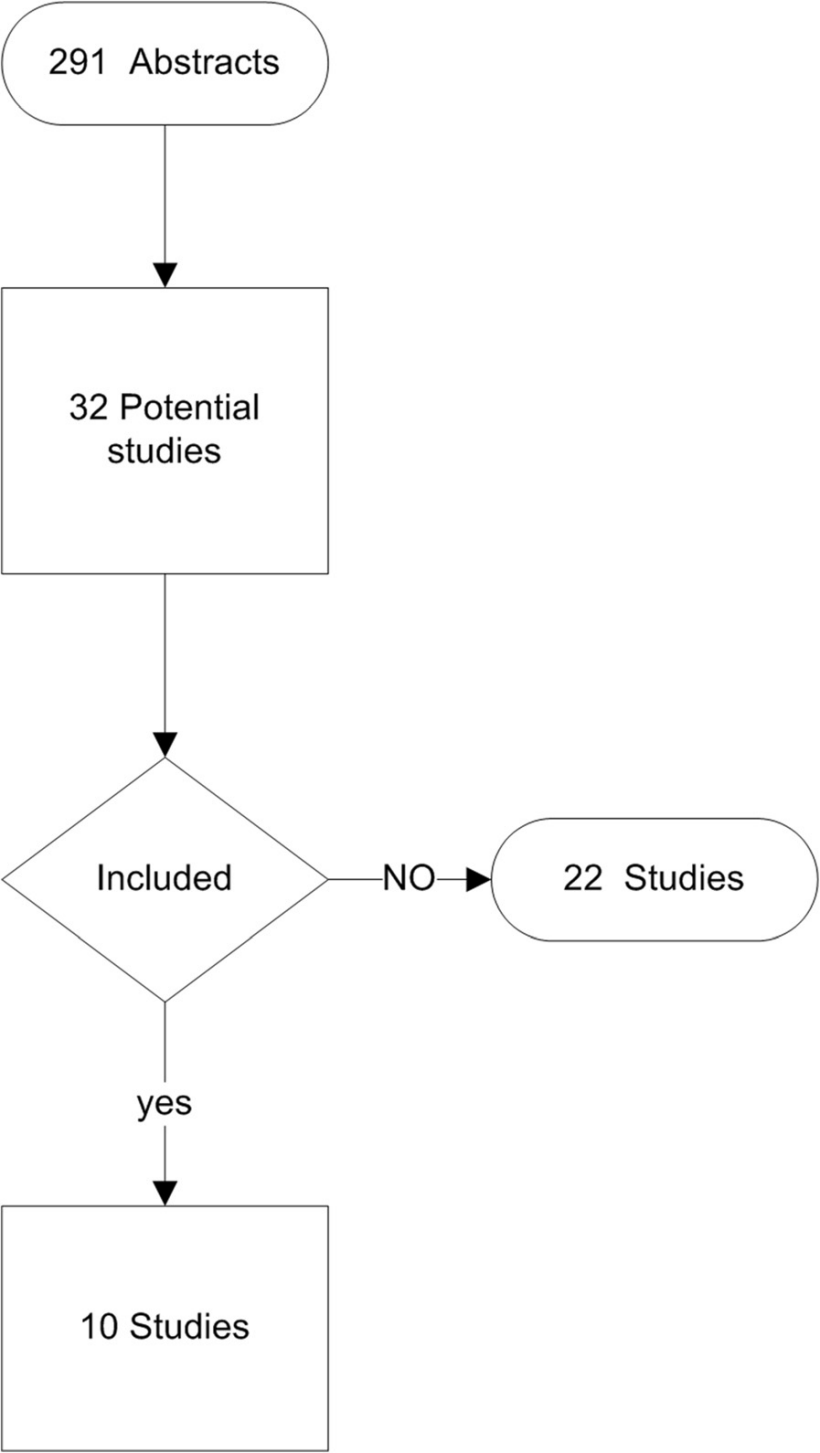
Flowchart describing selection of included studies

Of the 291 initially evaluated abstracts, 32 studies were initially selected. Twenty-two studies were subsequently excluded: ten studies did not evaluate patients [12–21], nine studies did not measure health literacy or did not use a validated instrument [22–30], and three did not evaluate patients in the perioperative setting [31–33] (Fig. 1). The characteristics of included studies are listed in Table 1. The studies evaluated included data from 1147 subjects in a variety of surgical procedures and were published between 2004 and 2013 [34–43]. The median (IQR) number of patients in the included studies was 101 (30 to 152). The majority of studies used the Short Test of Functional Literacy in adults (STOFHLA) to evaluate patients’ health literacy. Five studies evaluated the patients preoperatively [34–36, 40, 43], four studies evaluated patients in the postoperative period [37, 38, 41, 44], and in one study the time of evaluation in relation to the surgical procedure was not defined [39]. The lowest prevalence of inadequate health literacy was detected in kidney transplant patients, 6 out of 124 (5 %) [41], while the highest prevalence of inadequate health literacy was detected in orthopedic patients having total knee or total joint replacement, 86 out of 126 (60 %).

**Table 1:** Summary of studies included in analysis

Authors	Year of study	Surgical specialty	Number inadequate health literacy/total subjects	Health literacy instrument	Study design	Intervention	Outcome	Newcastle-Ottawa scale^a^ or modified Jadad score^b^
Chu et al. [34]	2013	Orthopedic	86/144	REALM	Case–control	None	Medical information comprehension	4^a^
Otal et al. [35]	2012	Pediatrics	30/79	Newest Vital Sign	Cross-sectional	None	Satisfaction with information	3^a^
Zite et al. [36]	2011	Gynecology	102/201	Chew’s screening items	Randomized controlled trial	Low literacy consent form	Consent form comprehension	3^b^
Choi et al. [37]	2011	Orthopedics	15/15	S-TOHFLA	Focus groups	Pictograph based discharge instructions	None	-
Beitler et al. [38]	2010	Ear Nose and Throat	3/8	S-TOHFLA	Cross-sectional	None	None	3^a^
Wallace et al. [39]	2009	Vascular	70/152	REALM	Cross-sectional	None	None	3^a^
Grubbs et al. [40]	2009	Transplant	14/62	TOHFLA	Cohort	None	Access to kidney transplant	5^a^
Gordon et al. [41]	2009	Transplant	6/124	S-TOHFLA/REALM-T	Cross-sectional	None	Kidney function	5^8^
Chew et al. [42]	2004	All ambulatory surgeries	40/332	S-TOHFLA	Cohort	None	Adherence to preoperative instructions	5^a^
Conlin et al. [43]	2002	Heart Surgery	6/30	REALM	Cross-sectional	None	Discharge Instruction comprehension	3^a^

^a^Newcastle-Ottawa scale

^b^Jadad score

One study developed and validated a rapid estimated of adult literacy specific for vascular surgery patients (Real_VS) [39]. The instrument had high internal consistency (Cronbachs α = 0.98) and high correlation with REALM scores (Spearman’s rank correlation = 0.91).

### Health literacy in the preoperative period

Five studies evaluated patients during the preoperative period [34–36, 40, 43]. Two studies (one a cross-sectional design and one case –control design) limited to evaluate the association of poor health literacy with information comprehension and information satisfaction [34, 35]. The study of Chu et al. [34] demonstrated using a validated questionnaire that patient comprehension of perioperative information was dependent on health literacy even after adjusting for provider’s empathy. In contrast, the study of Otal et al. [35] did not find an association between healthy literacy and patient satisfaction with perioperative information.

Two prospective cohort studies examined the association of health literacy and important perioperative outcomes [40, 43]. Chew et al. [42] evaluated the association between health literacy and adherence to preoperative medications in 332 ambulatory surgical patients. Despite observing a greater non-adherence to preoperative medications among patients with inadequate health literacy, the study was underpowered to detect a statistically significant difference, odds ratio (95 % CI) of 1.9 (0.8 to 4.8). Grubbs et al. [40] conducted a prospective cohort study to examine an association between health literacy and access to kidney transplant list among patients with chronic kidney failure. The authors found that subjects with inadequate health literacy were less likely to be referred to a transplant list, hazard ratio (95 % CI) of 0.22 (0.08 to 0.6), after adjusting for confounding factors such as age, gender, race and income.

Only one randomized trial evaluated the effect of low literacy consent on patient’s comprehension of the consent process in patients undergoing laparoscopic tubal ligation [36]. Women who were randomized to a low literacy consent form understood the consent process better than the women who were randomized to the standard consent form. However, the authors did not use a validated instrument to measure patient comprehension.

### Health literacy in the postoperative period

Four studies investigated the role of health literacy in the postoperative care of surgical patients [37, 38, 41, 44]. Three studies had a cross sectional design and one was based on discussions of focus groups.

One study evaluated the prevalence of inadequate health literacy in patients who underwent total laryngectomy [38]. The estimation was limited by the extremely small sample size (*n* = 8) and by a large proportion of patients lost to follow up.

One cross-sectional study evaluated the role of health literacy on patients ability to understand discharge instructions after cardiac surgery [44]. The authors detected a strong correlation between healthy literacy but not educational level with patients’ ability to comprehend discharge instructions (Pearson’s coefficient = 0.67). Another cross-sectional study evaluated the association between health literacy and creatinine levels in patients after kidney transplant [41]. The authors found a small but independent association between lower levels of health literacy and greater levels of creatinine (*β* = −0.3, 95 % CI −0.05 to −0.00; *P* = 0.03).

One author performed a focus group study to evaluate the effect of pictograph –based discharge instructions in patients that underwent hip replacement surgery [37]. Although the author concluded that pictograph based discharge instructions was an effective strategy to present discharge instructions for patients with inadequate health literacy, no formal analyses were presented by the author.

## Discussion

The most important finding of the current systematic review was the paucity of studies examining health literacy in the perioperative setting. In addition, current studies in surgical settings had very limited sample sizes especially when compared to studies in the medical setting [44, 45]. Several investigations were excluded because they did not even use a validated instrument to measure health literacy but often use educational level as a surrogate. Since healthy literacy has been associated with poor patient outcomes (including death) in the medical setting [5, 46], our systematic review establishes the extreme need for additional studies evaluating the role of health literacy in the perioperative setting.

Most of the included studies were observational and only one study examined a low-literacy patient communication intervention using a randomized controlled design [36]. The study was limited because the primary outcome (patient comprehension) was evaluated using a non-validated questionnaire. Doak et al. has provided a strategy through the Suitability of Assessment Material (SAM) to offer objective medical information in an appropriate manner to patients, especially those with limited health literacy [47]. In contrast, decision aids in the surgical setting have failed to adjust for different levels of healthy literacy among patients [48].

Health literacy is considered a major driving factor in explaining disparities in health care [49]. Multiple studies have evaluated disparities in surgical care but not the role of health literacy [50–52]. We were able to identify only one study that demonstrated a very strong association between poor health literacy and lack of patients’ enrollment on kidney transplant lists [40]. It is possible that health literacy may explain part of the currently described disparity in surgical services [53, 54]. Future studies attempting to explain disparities in surgical care should incorporate measurements of health literacy in their evaluation.

It was interesting to note that the reported rates of inadequate health literacy varied substantially among different surgical procedures. Poor health literacy had low prevalence in recipients of kidney transplants (4 %) but it had high prevalence among orthopedic patients having hip and knee replacements [34, 41]. Since poor health literacy has been repeatedly associated with advancing age [55, 56], it is possible that different age characteristics has contributed to the differences in inadequate health literacy among the evaluated surgical groups. Nevertheless, due to the relatively small sample sizes of included investigations, larger comprehensive studies are needed to establish which surgical specialties are most vulnerable to have patients with poor health literacy.

Currently, approximately 70 % of surgical procedures are performed in the ambulatory setting [57]. The ambulatory setting may be particular challenge for low literacy elderly patients that need hospital support but are sent home, irrespective of health literacy skills. In addition, discharge instructions are often given after surgery where cognitive function may be decreased due to anesthesia and analgesic medications [58, 59]. A recent study has demonstrated that age is an independent risk factor for the development of venous thrombosis after ambulatory surgery [60]. It remains to be determined if adequate health literacy skills are associated with a safer discharge of elderly ambulatory surgical patients.

Our systematic review should only be interpreted in the context of its limitations. Due to the low number of interventions and a variety of outcomes examined, we were unable to perform a quantitative analysis. Consequently, we were not able to examine the data for publication bias. We cannot, therefore, exclude the possibility of negative studies that were “file-drawer” and could have refuted the influence of inadequate health literacy on surgical outcomes. We were also not able to detect which levels of health literacy are critical for optimal outcomes in perioperative patients. The time constraints commonly seen in the surgical setting may require greater levels of health literacy from surgical patients than what has been established for optimal outcomes in the non-surgical specialties.

## Conclusions

In summary, we evaluated the role of health literacy for patient care in the perioperative setting. The evaluated studies suggest that poor health literacy may be associated with inadequate comprehension of the surgical procedure and discharge instructions. In addition health literacy may be implicated with poor adherence to preoperative instruction which may jeopardize patient safety. Lastly, surgical disparities may also be, in part, explained by inadequate literacy. The lack of large studies confirming those preliminary findings and the lack of interventions to address limited perioperative health literacy call for an extreme need to develop a research agenda in order to minimize the effects of poor health literacy in the care of surgical patients.
